# High National Institutes of Health Stroke Scale Scores, Hyperglycemia, and Anemia as Predictors of Length of Stay in Patients Hospitalized for Acute Ischemic Stroke

**DOI:** 10.7759/cureus.94792

**Published:** 2025-10-17

**Authors:** Hadi Abou-El-Hassan, Shayan Massoumi, Sara Akhlaghi, Reem Sarsour, Kathryn Pillai, Morgan Petersen, En Chang, Jonathan Kuo, Suhas Nareddy, Elias A Giraldo

**Affiliations:** 1 Department of Neurology, Arrowhead Regional Medical Center, Colton, USA; 2 Department of Neurology, California University of Science and Medicine, Colton, USA

**Keywords:** anemia, hyperglycemia, length of stay, nihss, stroke

## Abstract

Stroke is a leading cause of mortality and disability worldwide. However, little is known about the predictors of hospital length of stay (LOS) among stroke patients in community hospital settings. This study aimed to identify predictors of hospital LOS among patients admitted for acute ischemic stroke at a community hospital in Southern California. A retrospective chart review was conducted for patients hospitalized with acute ischemic stroke at Arrowhead Regional Medical Center between January 2024 and July 2024. Univariable and multivariable logistic regression analyses were performed to determine predictors of hospital LOS. Receiver operating characteristic (ROC) and area under the curve (AUC) analyses were used to assess the reliability of the logistic regression model. A total of 101 patient records were reviewed, of which 51% had a hospital stay longer than six days. On univariable logistic regression analyses, a total Glasgow Coma Scale score ≤12, National Institutes of Health Stroke Scale (NIHSS) score ≥10, initial glucose level ≥180 mg/dL, hemoglobin level ≤12 g/dL, and admission to the ICU were associated with prolonged hospital stay. On multivariable logistic regression analysis, only NIHSS ≥10, glucose ≥180 mg/dL, and hemoglobin ≤12 g/dL remained significantly associated with prolonged hospital stay. ROC/AUC analysis demonstrated good model reliability, with an AUC of 0.871. Initial clinical evaluation findings of NIHSS ≥10, glucose ≥180 mg/dL, and hemoglobin ≤12 g/dL were significant predictors of prolonged hospital stay among patients with acute ischemic stroke.

## Introduction

Ischemic stroke is a cerebrovascular event characterized by compromised blood supply to the central nervous system [1]. If this disruption is not promptly reversed, the resulting lack of blood flow can lead to irreversible infarction and loss of function in the affected cerebral tissue [2]. Acute ischemic stroke has various etiologies, including cardioembolism, large vessel disease, and small vessel disease [1]. The consequences of ischemic stroke include cognitive deficits and residual disability, which negatively affect quality of life and increase the risk of stroke recurrence [3].

Although the prevalence of ischemic stroke has declined due to improved management of cardiovascular risk factors, acute ischemic stroke still occurs frequently, with approximately 700,000 new or recurrent events reported annually in the United States [4]. The impact of ischemic stroke is profound, as it remains one of the leading causes of death worldwide and the primary cause of acquired long-term disability [5].

In the United States, the median length of stay (LOS) for ischemic stroke is six days, with a range from as short as one day to as long as 63 days [6]. Prolonged hospitalization may result in poor functional outcomes and unfavorable discharge dispositions, such as transfer to rehabilitation centers or nursing homes [7]. In a study comparing prolonged versus average hospital stays for acute ischemic stroke, 97% of patients in the prolonged-stay group were discharged in a severe functional state, with a modified Rankin Scale score of 4-5 [8]. Previously identified contributors to prolonged hospitalization include diabetes, advanced age, stroke severity, infarctions within the anterior cerebral circulation, and cardioembolic etiology [9].

Understanding the factors that influence LOS among patients hospitalized for acute ischemic stroke may help reduce hospital stay duration and improve patient outcomes. However, such factors remain largely understudied in community hospital settings. The purpose of this study is to identify predictors of hospital LOS among stroke patients admitted to a community hospital in Southern California. Specifically, we aim to determine whether patient demographics, comorbidities, metabolic abnormalities, in-hospital events, stroke burden, or other parameters can predict LOS among patients admitted for management of acute ischemic stroke.

## Materials and methods

Study design and patient selection

This was a retrospective observational study conducted at Arrowhead Regional Medical Center (ARMC), a community hospital in Southern California. The study reviewed medical records of patients admitted between January 1, 2024, and July 1, 2024.

The study population included all male and female patients aged 18 years and older who were admitted to ARMC with a diagnosis of acute ischemic stroke during the specified period. Patients were categorized into two groups based on their LOS: LOS ≤6 days and LOS >6 days.

Inclusion criteria were age >18 years, confirmed diagnosis of acute ischemic stroke, and hospital stay >24 hours. Exclusion criteria included age <18 years, hospital stay <24 hours, incarceration, pregnancy, and a history of traumatic brain injury or any prior brain surgery.

The sample size was calculated using the Cleveland Clinic online sample size calculator [10], based on the following formula:

\(n_1 = \frac{\left( z_{1-\alpha/2} \sqrt{(k+1)\bar{p}(1-\bar{p})} + z_{1-\beta} \sqrt{p_0(1-p_0) + k p_1(1-p_1)} \right)^2}{k(p_1 - p_0)^2}, \quad n_0 = k n_1,\)

$\text{where } \bar{p} = \frac{k p_0 + p_1}{k + 1}.$

Sample size was comparable to that of similar public health studies in the literature [8]. Data were collected from existing hospital records, and the study was approved by the Institutional Review Board of AMRC (approval number 24-08).

Data collection

Data were obtained through a retrospective review of medical records. The collected information included patient demographics, comorbidities, admission vital signs, metabolic abnormalities, significant in-hospital events (such as procedures, surgery, artificial feeding, and mechanical ventilation), acute stroke treatment, and stroke location. Data were recorded in a de-identified and encrypted spreadsheet. Patient identifiers were excluded from the analysis, and all data were aggregated prior to analysis.

Statistical analysis

Descriptive statistics for categorical variables were expressed as frequencies and percentages, while continuous variables were summarized as medians with first and third quartiles. Comparisons between the two groups were performed using one-tailed t-tests for continuous variables and χ² tests for categorical variables.

To identify variables associated with outcomes and adjust for potential confounders, both univariable and multivariable logistic regression models were employed, from which ORs and 95% CIs were derived. Missing data were excluded from the analysis. Receiver operating characteristic (ROC) and area under the curve (AUC) analyses were used to evaluate the predictive performance of the logistic regression model. The AUC was interpreted as follows: 0.7 to <0.8 indicated acceptable discrimination, and 0.8 to <0.9 indicated excellent discrimination.

All statistical analyses were performed using R version 4.2.2, with a statistical significance level set at p < 0.05.

## Results

A total of 101 patients with acute ischemic stroke who met the study criteria were identified. Baseline characteristics are presented in Table 1. Forty-nine patients had an LOS ≤6 days, while 52 patients had an LOS >6 days. The median LOS was 4 days (IQR: 3-5) in the ≤6 days group and 17 days (IQR: 11-37) in the >6 days group.

**Table 1:** Characteristics of the study subjects ¹ Median (Q1, Q3); n (%) ² Two-sample t-test for continuous variables; Pearson’s chi-squared test for categorical variables GCS, Glasgow Coma Scale; HbA1c, hemoglobin A1c; LDL, low-density lipoprotein; LOS, length of stay; MAP, mean arterial pressure; NIHSS, National Institutes of Health Stroke Scale; SBP, systolic blood pressure; TIA, transient ischemic attack

Characteristic (n = 101)	LOS ≤6 days (n = 49)¹	LOS >6 days (n = 52)¹	p-Value²
LOS	4 (3, 5)	17 (11, 37)	-
Age	68 (61, 76)	67 (55, 76)	0.6
Sex			0.53
Female	15 (30.6%)	19 (36.5%)	
Male	34 (69.4%)	33 (63.5%)	
Race			0.68
White	12 (24.5%)	17 (32.7%)	
African American	5 (10.2%)	8 (15.4%)	
Hispanic	27 (55.1%)	22 (42.3%)	
Asian	2 (4.1%)	3 (5.8%)	
Other	3 (6.1%)	2 (3.8%)	
BMI (kg/m²)	29.0 (26.0, 34.6)	28.7 (24.5, 33.2)	0.17
Hypertension	41 (83.7%)	38 (73.1%)	0.2
Diabetes mellitus	19 (38.8%)	24 (46.2%)	0.45
Hyperlipidemia	21 (42.9%)	20 (38.5%)	0.65
Atrial fibrillation	8 (16.3%)	9 (17.3%)	0.9
History of TIA	10 (20.4%)	19 (36.5%)	0.07
Alcohol	10 (20.4%)	7 (13.5%)	0.35
Smoking	15 (30.6%)	13 (25.0%)	0.53
Substance use	4 (8.2%)	7 (13.5%)	0.39
Admission SBP (mmHg)	157 (136, 179)	146 (132, 174)	0.97
Admission MAP (mmHg)	107 (92, 124)	108 (92, 125)	0.59
Total GCS	15.00 (14.00, 15.00)	13.50 (10.00, 15.00)	0.02
NIHSS	6 (3, 9)	9 (6, 15)	0.03
Glucose (mg/dL)	122 (103, 159)	152 (113, 204)	0.05
WBC (× 10⁹/L)	8.6 (6.8, 11.3)	9.5 (7.0, 12.3)	0.09
Hemoglobin (g/dL)	13.80 (12.60, 15.10)	13.95 (11.20, 15.70)	0.52
Platelets (× 10⁹/L)	235 (191, 288)	239 (182, 316)	0.39
Sodium (mmol/L)	138 (135, 140)	139 (136, 141)	0.52
CO₂ (mmol/L)	24 (23, 26)	23 (21, 25)	0.45
HbA1c (%)	6.20 (5.60, 7.00)	6.65 (5.75, 6.93)	0.77
LDL (mg/dL)	98 (71, 121)	98 (67, 122)	0.84
Acute stroke			0.52
Anterior	37 (75.5%)	42 (80.8%)	
Posterior	12 (24.5%)	10 (19.2%)	
Thrombolysis	11 (22.4%)	5 (9.6%)	0.08
Antiplatelet therapy	31 (63.3%)	27 (51.9%)	0.25
Thrombectomy	3 (6.1%)	6 (11.5%)	0.49
Admit to			0.03
Ward	35 (71.4%)	26 (50.0%)	
ICU	14 (28.6%)	26 (50.0%)	
Nasogastric tube	8 (16.3%)	20 (38.5%)	0.01
Seizure	2 (4.1%)	3 (5.8%)	1
Infection	5 (10.2%)	11 (21.2%)	0.13
Discharged to			0.09
Home	21 (42.9%)	12 (23.1%)	
Acute rehabilitation	10 (20.4%)	8 (15.4%)	
Skilled nursing facility	7 (14.3%)	14 (26.9%)	
Long-term acute care	0 (0.0%)	2 (3.8%)	
Other	11 (22.4%)	16 (30.8%)	

Patients in the two groups were similar in terms of median age and male-to-female ratio. Most patients were Hispanic (55.1% in the LOS ≤6 days group vs. 42.3% in the LOS >6 days group), consistent with the demographic composition of the ARMC patient population. There were no significant differences in the prevalence of comorbidities between the two groups.

Upon initial assessment, patients with an LOS >6 days had a lower Glasgow Coma Scale (GCS) score (15.0 vs. 13.5, p = 0.02) and a higher National Institutes of Health Stroke Scale (NIHSS) score (6 vs. 9, p = 0.05). Among the initial laboratory findings, patients with an LOS >6 days also had higher blood glucose levels (122 vs. 152 mg/dL, p = 0.05).

Regarding acute management, patients with an LOS ≤6 days more frequently received chemical thrombolysis (p = 0.08), whereas those with an LOS >6 days were more often admitted to the ICU (p = 0.03) and underwent nasogastric tube placement (p = 0.01). Patients with an LOS >6 days also more frequently developed infections, although this finding was not statistically significant (p = 0.13).

Using univariate logistic regression analysis, we identified independent predictors of a LOS >6 days (Table 2). Total GCS ≤ 12 (OR 3.20, 95% CI 1.29-8.61, p = 0.015), NIHSS ≥10 (OR 2.53, 95% CI 1.08-6.20, p = 0.036), initial glucose ≥180 mg/dL (OR 2.44, 95% CI 1.02-6.13, p = 0.050), hemoglobin ≤ 12 g/dL (OR 2.91, 95% CI 1.06-8.85, p = 0.045), ICU admission (OR 2.50, 95% CI 1.11-5.88, p = 0.029), and nasogastric tube placement (OR 3.20, 95% CI 1.29-8.61, p = 0.015) were all associated with an LOS exceeding 6 days.

**Table 2:** Univariable logistic regression analysis for LOS >6 days GCS, Glasgow Coma Scale; LOS, length of stay; MAP, mean arterial pressure; NIHSS, National Institutes of Health Stroke Scale; SBP, systolic blood pressure; TIA, transient ischemic attack

Independent predictor	OR (95% CI, p-Value)
Age	0.99 (0.96-1.02, p = 0.594)
Sex, male	0.77 (0.33-1.75, p = 0.529)
Race, White	0.89 (0.22-3.35, p = 0.859)
Race, Hispanic	0.51 (0.14-1.75, p = 0.290)
Race, Other	0.42 (0.04-3.39, p = 0.416)
BMI ≥30 kg/m²	0.81 (0.37-1.77, p = 0.599)
History of TIA	2.25 (0.93-5.66, p = 0.077)
Admission SBP ≥140 mmHg	0.65 (0.28-1.47, p = 0.307)
Total GCS ≤ 12	3.20 (1.29-8.61, p = 0.015)
NIHSS ≥10	2.53 (1.08-6.20, p = 0.036)
Glucose ≥180 mg/dL	2.44 (1.02-6.13, p = 0.050)
WBC ≥ 12 × 10⁹/L	1.80 (0.71-4.76, p = 0.219)
Hemoglobin ≤12 g/dL	2.91 (1.06-8.85, p = 0.045)
Acute stroke, posterior	0.73 (0.28-1.90, p = 0.523)
Thrombolysis	0.37 (0.11-1.10, p = 0.085)
Antiplatelet therapy	0.63 (0.28-1.38, p = 0.250)
Thrombectomy	2.00 (0.50-9.93, p = 0.347)
Admit to ICU	2.50 (1.11-5.88, p = 0.029)
Nasogastric tube	3.20 (1.29-8.61, p = 0.015)
Seizure	1.44 (0.23-11.29, p = 0.697)
Infection	2.36 (0.79-8.03, p = 0.140)

In multivariable logistic regression analysis, NIHSS ≥10 (adjusted OR 6.80, 95% CI 1.43-41.84, p = 0.024), initial glucose ≥180 mg/dL (adjusted OR 7.27, 95% CI 1.85-35.39, p = 0.007), and hemoglobin ≤ 12 g/dL (adjusted OR 5.74, 95% CI 1.27-33.14, p = 0.032) remained significant predictors of an LOS >6 days.

**Figure 1:**
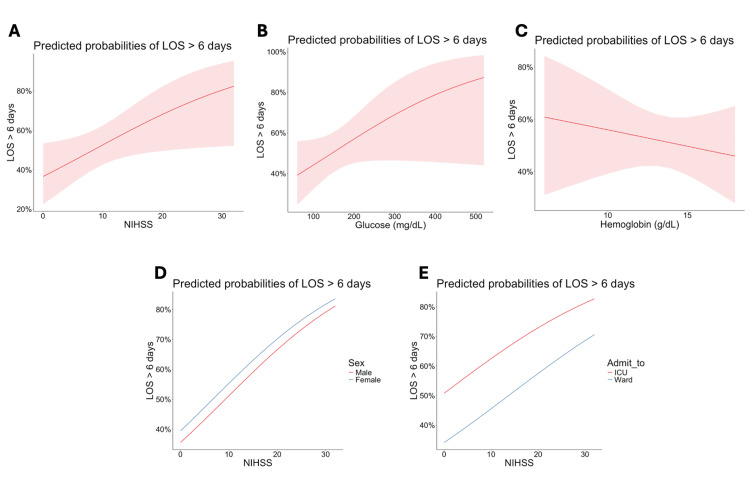
Predicted probabilities of LOS >6 days according to independent variables: NIHSS, glucose, and hemoglobin (A) Predicted probabilities by NIHSS score. (B) Predicted probabilities by initial glucose level. (C) Predicted probabilities by initial hemoglobin level. (D) Predicted probabilities by NIHSS score stratified by sex. (E) Predicted probabilities by NIHSS score stratified by admission unit. LOS, length of stay; NIHSS, National Institutes of Health Stroke Scale

**Figure 2:**
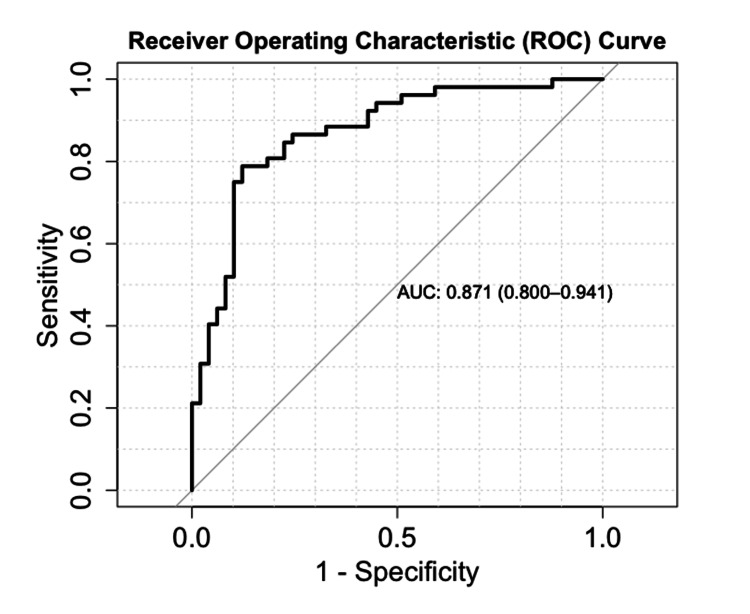
ROC curve of the logistic regression model AUC, area under the curve; ROC, receiver operating characteristic

We tabulated the predicted probability of an LOS >6 days based on NIHSS scores, glucose levels, and hemoglobin levels (Figure 1). In Figure 1A, the probability of a hospital stay exceeding six days increases as the NIHSS score rises. In Figure 1B, initial glucose levels >180 mg/dL are associated with a higher probability of an LOS >6 days. In Figure 1C, the probability of prolonged LOS increases as hemoglobin levels decrease. Figure 1D shows no significant difference in prolonged LOS between males and females. Figure 1E demonstrates that, for a given NIHSS score, patients admitted to the ICU have a higher probability of an LOS >6 days compared with those admitted to the general floor. ROC analysis indicates that the logistic regression model reliably predicts LOS >6 days, with an AUC of 0.871 (Figure 2).

## Discussion

In this retrospective cohort study of patients admitted for acute ischemic stroke at ARMC, we investigated predictors of hospital LOS. LOS was analyzed as a categorical variable, dividing patients into two groups: those with LOS ≤6 days and those with prolonged LOS >6 days. Baseline characteristics showed that patients with prolonged stays had significantly lower total GCS scores, higher NIHSS scores, higher rates of ICU admission, higher blood glucose levels, and more frequent need for nasogastric tube placement (Table 1). This pattern is also illustrated in Figure 1A-1C, where increasing NIHSS scores, elevated glucose levels, and decreased hemoglobin levels predicted a higher probability of prolonged LOS. Figure 1D, 1E presents subgroup analyses for NIHSS scores, showing no significant difference in prolonged LOS between sexes, whereas ICU admission was associated with a significantly higher likelihood of prolonged LOS. These findings were further supported by univariate and multivariate regression analyses, which identified NIHSS ≥10, initial blood glucose >180 mg/dL, and hemoglobin ≤ 12 g/dL as significant predictors of LOS ≥6 days (Table 2).

Our results align with prior literature showing that patients with NIHSS scores >10 are more likely to experience prolonged hospitalization [11]. Stroke severity, as measured by the NIHSS, is a well-established predictor of LOS [12]. Higher NIHSS scores reflect more severe neurological deficits, which likely contribute to longer hospital stays due to the need for intensive monitoring, extended medical stabilization, and multidisciplinary evaluation by physical and speech therapists prior to safe discharge.

The observed association between initial blood glucose levels >180 mg/dL and prolonged LOS is also consistent with previous studies, which indicate that hyperglycemia on admission correlates with worse functional outcomes and higher complication rates in stroke patients [13]. This may partly reflect the high prevalence of diabetes mellitus in this population, where diabetes-related complications can worsen recovery [14]. Importantly, even in patients without preexisting diabetes, acute hyperglycemia has been linked to poorer functional recovery and higher in-hospital mortality [15]. Mechanistically, hyperglycemia may increase oxidative stress and disrupt the blood-brain barrier, directly damaging tight junction proteins, making the brain more susceptible to vasogenic edema [16]. Consequently, ischemic stroke patients may face complications such as worsening ischemia and hemorrhagic transformation, necessitating prolonged monitoring and intervention, which can extend LOS. Hyperglycemia also increases susceptibility to infections, such as pneumonia and urinary tract infections, further prolonging hospitalization. In our cohort, we observed a higher rate of infections among patients with LOS >6 days; however, this difference did not reach statistical significance, likely due to the modest sample size, a limitation of our study.

Similarly, hemoglobin levels ≤12 g/dL were associated with prolonged LOS, likely due to reduced oxygen delivery to ischemic brain tissue, which can exacerbate neuronal injury, slow recovery, and increase the need for inpatient care. Previous studies have also linked anemia with larger infarct sizes [17], suggesting that patients with baseline hemoglobin ≤12 g/dL may be more prone to severe neurological deficits, necessitating extended hospitalization.

Limitations

This study has several limitations. The population was predominantly Hispanic and located in southern California, limiting generalizability. Larger, multicenter studies are needed to determine whether these findings apply to broader populations. The sample size was constrained by the number of patient records available for review, which may limit statistical power. Additionally, some potentially relevant variables, such as smoking, alcohol use, and substance use, were not consistently documented, which may influence LOS. Future studies should also explore whether targeted interventions, such as glucose management protocols or early anemia treatment, could reduce LOS in this patient population.

## Conclusions

Our findings underscore the importance of early multidisciplinary intervention and resource allocation for patients with high NIHSS scores, hyperglycemia, and anemia to potentially reduce unnecessary hospitalization and associated costs. Given that ischemic stroke is a leading cause of death worldwide and the primary cause of long-term disability, identifying modifiable factors influencing LOS can optimize in-hospital stroke care. Recognizing independent predictors of prolonged hospitalization highlights opportunities for targeted interventions to streamline stroke management. If corroborated in larger studies, these findings could improve hospital workflow, reduce healthcare costs, and enhance patient outcomes.
